# Posttraumatic Stress among Not-Exposed Traumatically Bereaved Relatives after the MS *Estonia* Disaster

**DOI:** 10.1371/journal.pone.0166441

**Published:** 2016-11-28

**Authors:** Josefin Sveen, Lilian Pohlkamp, Joakim Öhlén, Jonas Sandberg, Kristina Brandänge, Petter Gustavsson

**Affiliations:** 1 Palliative Research Centre, Ersta Sköndal University College, Stockholm, Sweden; 2 Department of Neuroscience, Psychiatry, Uppsala University, Uppsala, Sweden; 3 Department of Neurobiology, Care Science and Society, Karolinska Institutet, Stockholm, Sweden; 4 Institute of Health and Care Sciences, and University of Gothenburg Centre for Person-Centered Care, University of Gothenburg, Gothenburg, Sweden; 5 Department of Nursing Science, School of Health and Welfare, Jönköping University, Jönköping, Sweden; 6 Department of Psychiatry, Ersta Hospital, Stockholm, Sweden; 7 Division of Psychology, Department of Clinical Neuroscience, Karolinska Institutet, Stockholm, Sweden; Technion Israel Institute of Technology, ISRAEL

## Abstract

**Background:**

Little is known about posttraumatic stress (PTS) reactions in bereaved individuals following loss in disaster who were not directly exposed to disaster. The aim of the present study was to examine the course of PTS up to three years after losing relatives in the MS *Estonia* ferry disaster, one of the worst maritime disasters in modern times.

**Methods:**

Seven postal surveys were sent out over three years post-disaster. The respondents were invited and added consecutively during the three years and 938 relatives participated in one or more of the surveys, representing 89% of the MS *Estonia*’s Swedish victims. The survey included the Impact of Event Scale (IES) to measure PTS. Latent growth curve modeling was used to analyze PTS over time.

**Results:**

The majority of bereaved individuals had high levels of PTS. At three years post-loss, 62% of the respondents scored above the recommended cut-off value on the IES. Over time, PTS symptoms declined, but initially high symptoms of PTS were associated with a slower recovery rate.

**Conclusion:**

The present finding suggests that being an indirectly-exposed disaster-bereaved close-relative can lead to very high levels of PTS which are sustained for several years.

## Introduction

The unexpected and sudden loss of a family member in disaster is one of the most traumatic events a person can experience and often leads to negative long-term effects on mental health [1]. Moreover, traumatic loss has been suggested to be more detrimental than normal bereavement [1]. High rates of posttraumatic stress (PTS) symptoms have been reported in individuals bereaved by disaster [2–4], however the prevalence of posttraumatic stress disorder (PTSD) following this type of traumatic loss varies [5].

Some studies have examined the longitudinal course of PTS in disaster survivors [6]. A recent study on the trajectories of PTS found that 16% of individuals displayed chronic PTS six years after a natural disaster [7] and the loss of a family member was a strong predictor of long-term PTS. Arnberg, Eriksson [8] reported findings from a longitudinal follow-up of survivors of the MS *Estonia* disaster where 27% experienced significant PTS after 14 years. PTS reactions declined during the first year after the disaster, but only minor changes were found between 1 year and 14 years afterwards. Traumatic bereavement was associated with worse long-term outcome. The difference in long-term PTS between bereaved and non-bereaved survivors appeared to arise from a recovery from the PTS reactions by non-bereaved survivors during the first year, but there was little change in the reactions of the bereaved survivors. These findings, as well as other research [9, 10], show that when PTS symptoms are not resolved within the first few years there is a risk of long-term problems for those exposed to disaster, which is in accordance with the theory of PTSD maintenance [11].

There is a lack of longitudinal studies investigating PTS reactions in bereaved individuals who have not been directly exposed to disaster. Johannesson, Lundin [4] found that one-third of those individuals who were not directly exposed but who were bereaved by the 2004 Indian Ocean Tsunami had high levels of PTS symptoms 21 months post-loss. In a study of Norwegian tsunami-bereaved individuals, 2% had PTSD 2 years post-loss and none 6 years post-loss for those not directly exposed, however, these estimates are uncertain as they are based on a small sample (*n* = 66) [12].

In 1994, the passenger ferry MS *Estonia* was shipwrecked on its way from Tallinn to Stockholm and, out of 989 persons on board, a mere 137 individuals survived, leaving a vast number of traumatically bereaved relatives. Symptoms of general psychological distress have previously been reported from this sample, but not PTS symptoms [13]. The aim of the present study was to examine the course of PTS reaction from three months (in intervals of 6 months) up to three years after losing a relative in the MS *Estonia* ferry disaster and to examine possible contributing factors with regards to sociodemographic characteristics, multiple losses and whether there was a missing body.

## Methods

### Procedure and sample

Of the 989 persons on board the MS *Estonia*, 552 were Swedish passengers. Of them, 501 died and only 40 bodies were found [14]. A public announcement was made four days after the disaster by the Ersta Psychiatric Clinic in Stockholm, offering psychological support activities to relatives of the disaster victims. It was decided that a questionnaire would be issued to those relatives affected by the disaster, in addition to inviting them to regular debriefing groups and official meetings.

Thus, a postal survey was sent out three months post-disaster (T1) to each and every estate of the deceased in Sweden. A second survey was sent out six months post-disaster (T2), and five subsequent surveys were sent out every six months (T3-T7), thus, in total, 7 surveys were sent out over the three years post-disaster. From the second survey, respondents were asked to invite other members of the estates to participate. Thus, additional respondents were invited and added consecutively during the three years. An eighth survey was conducted 5.5 years post-disaster, but its results are not included in the present paper.

In total, 938 relatives participated in one or more of the surveys, representing 89% of the MS *Estonia*’s Swedish victims. The participants were 48% women and 52% men. The majority of the respondents (60%) were 30–60 years of age at the time of the disaster, with a mean age of 45 (SD = 17). A total of 113 individuals suffered multiple losses. Sub-groups used in this study included individuals who experienced loss of children of all ages (*n* = 188), partners (*n* = 173), siblings (*n* = 171) and parents (*n* = 361). Additionally, individuals suffering losses of other relationships also existed, i.e. close friends and significant others, but these were not sub-grouped and were only included in the total sample. The number of respondents at each survey varied between 400 and 483 individuals, and 682 respondents participated in two or more surveys. The study was approved by the Regional Ethics Review Board in Stockholm, Sweden (approval: 22/95, 359/98, 2012/1209-32).

### Measurements

#### Survey instrument

The surveys included questions about the participants’ demographics, specific questions regarding what had happened in the last three months, a symptom checklist, and the Impact of Event Scale (IES). In this paper the IES and its demographic variables have been used.

*The Impact of Event Scale (IES)* [15] was used to assess symptoms of PTS. It contains 15 items divided into two sub-scales: Intrusion (7 items) and Avoidance (8 items). The items are rated on a 4-point Likert-type scale: 0, 1, 3, and 5, where 0 equals no symptom and 5 equals a high frequency of the symptom during the past week pertaining to a specific event. Total scores range from 0 to 75 and are achieved by summing all items. The Swedish version performed best as a screening measure for PTSD, according to the diagnostic criteria in the fourth version of the Diagnostic and Statistical Manual of Mental disorders (DSM-IV) [16], with a cut-off score of 25 (discriminant ability = 0.83) [17]. The internal consistency for the total IES scales varied between 0.85–0.93 over time.

### Statistical analysis

Data analysis was performed using IBM SPSS version 21 for Windows. The proposed IES cut-off value (>25) was used to describe the percentage of participants with self-reported symptoms of PTS. The original scoring of the IES, i.e., 0, 1, 3 and 5, was used in the analysis to make it comparable to other studies using the IES. Prevalence was computed for the total sample, as well as for the following sub-groups; loss of child, partner, sibling, parent, and multiple losses and missing body, at each time point.

The longitudinal analysis of PTS applied the multilevel model (also called the linear mixed model) for change, implemented as the latent growth curve modeling in the structural equation modelling framework [18]. Growth curve modeling can be used to estimate a linear trajectory for the entire sample, that is: estimating an intercept and a slope describing the changes in PTS reactions over time; concurrently estimating the influence of a latent factor explaining individual variability in initial levels of PTS; and a latent factor explaining individual variability in the rate of change across time. Two fixed effects (intercept and slope) as well as variance (and covariance) around these parameters (i.e. three random effects) were estimated using Full Information Maximum Likelihood (FIML) in Mplus 7.1 [19]. Model fit was evaluated using recommendations based on simulations [20]. Specifically, good model fit was indicated by a standardized root mean square residual (SRMR) of below 0.08, a root mean square error of approximation (RMSEA) of around 0.05, and a comparative fit index (CFI) of around 0.95.

## Results

The IES total mean scores were high at each of the seven time-points and the proportion of individuals who scored above the cut-off value was also very high for the total group, as well as for the subgroups (Table 1). The highest IES mean scores in the total group were found three months post-loss with subsequently lower scores over time. Among the sub-groups, the highest IES mean scores were reported among those who had lost their child. In this group the IES mean levels were around 45 across all time-points and the proportion of individuals who scored above the cut-off value was highest at 3 months (96%), and lowest at 12 months (83%), both of which were substantially higher than the scores for the total group or for any other sub-group at the same time-point.

**Table 1:** Impact of Event Scale total mean scores and proportion above cut-off score from 3 months up to 36 months post-loss.

	3 mo			6 mo			12 mo			18 mo			24 mo			30 mo			36 mo		
	*n* = 447			*n* = 483			*n* = 410			*n* = 483			*n* = 426			*n* = 400			*n* = 451		
	M	SD	% >25	M	SD	% >25	M	SD	% >25	M	SD	% >25	M	SD	% >25	M	SD	% >25	M	SD	% >25
Total sample (*n* = 938)	40.8	8.2	83.0	38.8	9.6	76.3	39.0	11.6	71.5	37.9	10.4	69.1	37.8	10.6	69.0	36.5	11.1	63.4	35.9	11.3	62.0
*Subsamples*																					
Loss of child (*n* = 188)	45.6	11.9	95.8	44.6	14.8	91.2	43.2	17.3	82.7	43.1	15.8	84.9	45.9	14.6	89.0	46.4	16.3	88.6	45.3	16.5	90.2
Loss of partner (*n* = 173)	40.6	13.3	84.4	37.3	14.9	75.0	35.5	16.5	69.0	36.7	17.5	69.5	35.4	19.1	63.2	33.8	18.6	61.5	30.0	19.2	56.8
Loss of sibling (*n* = 171)	40.5	15.1	79.7	38.0	15.9	76.2	36.1	16.2	76.6	36.2	17.1	73.2	34.9	16.3	69.4	29.4	17.8	52.5	32.1	18.2	60.3
Loss of parent (n = 361)	39.6	14.6	78.4	34.9	16.2	70.9	32.9	16.0	65.0	31.6	17.3	57.6	32.4	17.3	62.7	29.4	17.2	56.6	27.6	17.3	51.8
Multiple losses (n = 113)	38.7	13.5	83.1	37.2	14.2	75.9	38.5	15.5	78.9	36.1	17.6	68.6	37.9	18.7	72.7	33.9	17.2	73.5	30.2	18.6	53.6
Missing body (*n* = 824)	41.0	8.3	80.4	38.7	9.5	78.9	39.0	11.7	70.0	38.0	10.3	73.3	38.3	10.6	60.6	36.8	11.1	63.3	36.5	11.2	50.0

mo = months; M = mean; SD = standard deviation, % = percentage of participants scoring above cut-off score on IES

**Fig 1:**
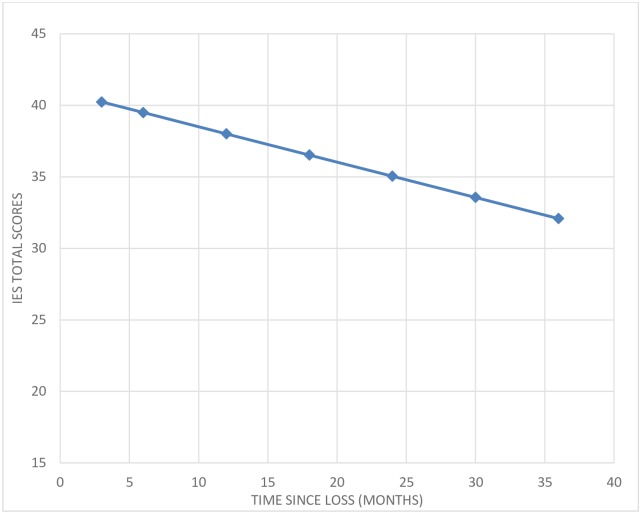
Longitudinal model describing linear change of Impact of Event Scale total mean scores. *Note*. *N* = 900; Χ^2^ = 51.18; df = 23; p = 0.0005; Root Mean Square Error of Approximation = 0.038; = Comparative Fit Index = 0.981; SRMR = 0.52; Intercept = 40.23; Slope = -1.482, Variance intercept = 175.566; Variance slope = 3.083.

A longitudinal model describing linear change was evaluated and showed good fit to the data. The general trend showed that IES total scores decreased over time (slope = -1.491; *p*<0.001). Thus, after every six months, there was a decrease in IES scores by approximately 1.5 points. The longitudinal trend is depicted in Fig 1. Individual differences around intercept and slope were statistically significant, indicating that individuals with initial lower scores showed a steeper decrease in PTS over time (covariance = 5.999; *p*<0.01).

**Fig 2:**
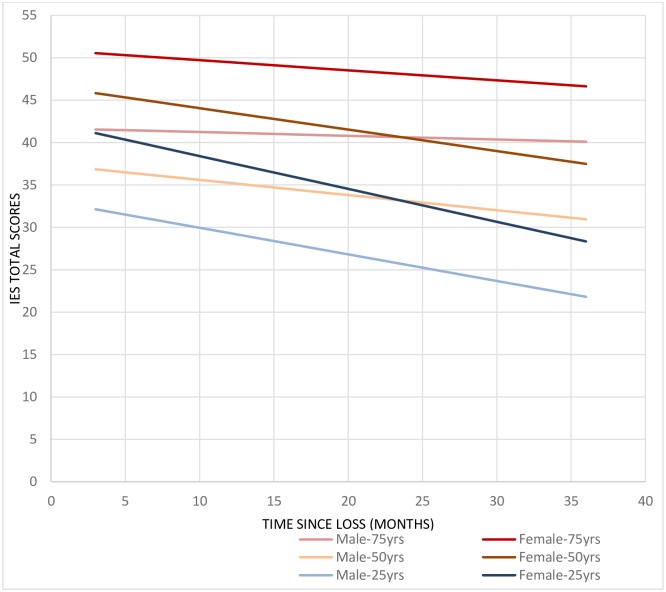
Longitudinal model describing linear change of Impact of Event Scale total mean scores, estimated trajectories are shown for different combinations of age and sex.

The model that included possible contributing factors showed that age and gender influenced the PTS reactions (see Table 2). At baseline, women’s IES scores were higher than men’s (*p*<0.001) and older participants had, in general, higher IES scores (*p*<0.001). Moreover, older respondents recovered at a slower rate compared to younger respondents, who showed a steeper decrease in IES scores over time (see Table 2). In addition, men recovered at a slower rate, compared to women who showed a steeper decline. In Fig 2, model-estimated trajectories are shown for different combinations of age and sex. To have lost more than one person and whether the body was found, did not affect the baseline scores or rate of change over time.

**Table 2:** Prediction of posttraumatic stress. Associations between four predictors and individual differences in baseline IES scores and rate of change in IES scores given as standardized regression coefficients (β) taken from latent growth model (*n* = 857, df = 43).

Predictors	Baseline (β)	*p*	Rate of change (β)	*p*
Age	0.242	<0.001	0.309	<0.001
Gender	0.338	<0.001	-0.125	0.040
Multiple losses	-0.039	0.254	-0.005	0.936
Missing body	-0.028	0.249	-0.112	0.249

## Discussion

The present longitudinal study demonstrates high levels of self-reported PTS in disaster-bereaved individuals, who were not directly exposed to the disaster. The majority of bereaved individuals had high levels of PTS. At three years post-loss, 62% of the respondents scored above cut-off value, and 90% of the parents who had lost a child. Longitudinal results revealed that high PTS levels declined slightly over time and that initially high symptoms of PTS were associated with a slower recovery rate. Yet, women had initially higher levels of PTS and recovered faster than men. In addition, older individuals recovered more slowly than those of younger age.

The persistence of high symptom levels is in line with the theoretical account of the maintenance of PTSD [11], as mentioned in the introduction, that individuals with PTS reaction that is not resolved within the first few years have a greater risk of experiencing sustained psychological distress [8–10, 21, 22]. Although there was a linear decrease in scores over time, the decrease was slow and, on a group level, the scores for those who had lost a child did not decrease from three months to three years post-loss. In addition, previous studies have shown that the loss of a child is more detrimental than the loss of other close-relatives [4, 12, 23], findings which are in accordance with the present study.

The traumatically bereaved individuals in this study appear to have suffered more than has been seen in other studies of those not directly exposed [4, 12]. For example, Kristensen, Weisaeth [12] used the structured diagnostic interview tool, the Mini International Neuropsychiatric Interview, to measure PTSD, and found that only three out of 66 not-directly-exposed disaster-bereaved individuals had PTSD two years post-loss and none had PTSD at six years post-loss. The reason for this difference needs further investigation.

Previous studies have shown that women have a higher risk of developing PTSD [24]. In the present study, women had initially higher scores, but the rate of decrease of their scores was somewhat steeper, thus women initially showed more PTS reactions but they recovered faster. This finding is similar to that of a study of the survivors of the Buffalo Creek flooding [25], which found that women’s mental health was initially worse than men, but after 14 years, results for both genders were the same. Likewise, in a study of PTSD after motor vehicle accidents, it was found that women had more PTSD than men after one month, and that after 6–12 months there was no difference [26, 27]. Moreover, age had an effect on the results in the present study; older individuals recovered more slowly than those of younger age. According to a meta-analysis of risk factors for PTSD, age has been found to predict PTSD in some populations, but not in others [24].

Not being able to recover the body did not have an impact on the psychological distress in the present study. However, this could be due to the fact that most bodies were missing (88%) and thus the results were not significant. A tentative explanation for the high levels of PTS in the present sample is that most relatives had to grieve without a body which may disrupt the grieving process and PTS reactions, as not viewing the body may make the loss unreal and disrupts the “continuing bond” that the bereaved maintains with the deceased [28]. Another tentative explanation for the high levels of PTS is that they were influenced by the knowledge that it was one of the worst maritime disasters in modern history and, in addition, on a societal level, the post-event exposure was exceptional, including substantial media debates and official investigations [29]. Thus, the participants were continuously exposed to the event and they could not mourn in peace and quiet.

Multiple-loss was not a predictor of worse bereavement outcome in the present study. This may be interpreted as being that the number of losses did not have a cumulative effect on the bereavement outcome, but instead, in the present study, it was the type of loss, i.e. the loss of a child, which had a stronger effect.

The results display necessities for providing appropriate support for disaster-bereaved individuals to possibly reduce their PTS and prevent prolonged PTSD, especially for bereaved parents as they are a high-risk group for worse bereavement outcome. In this way there is a need for support that is tailored to respond to the special loss for disaster-bereaved people as compared to loss following illness and diseases [5]. However, systematic reviews display a lack of evidence for specific advice and supportive interventions for disaster-bereaved persons, including those for disaster-bereaved parents [30].

Fortunately, catastrophes such as the MS *Estonia* disaster are not frequent. Still, the reality of these unexpected events necessitates the need to have structures in readiness to put supportive plans into action when needed; on societal, community and family network levels. Given the vast number of types of events leading to sudden and traumatic deaths (single cases, small- and large-scale) that could imply loss with disaster experience [31, 32], there is a continuous need to provide support to disaster-bereaved individuals in every society. In particular, the initially high and long-lasting levels of PTS presented in this study indicate a need to include loss from disaster as a mental health issue.

There are several limitations of the study, including the recruitment process, as some participants were recruited through other participants within the same estate and there are no data available for non-responders. However, a strength of the study is that the respondents make up 89% of the relatives of the Swedish deceased. Another limitation is that there are no data regarding the respondents’ socioeconomic status and limited information regarding their sociodemographic variables. A further limitation is the reliance of self-reported measures of PTS; nevertheless, the IES is one of the most widely used PTS measures and its validity has been tested in several studies [33]. A limitation of the study is that since the data collection was initiated in 1994, the diagnostic criteria of PTSD have changed and thus the use of IES could be considered outdated, hence the proportion of individuals scoring above the recommended cut-off value may not be an indication of PTS cases. Some of the participants received support and/or debriefing, which may have influenced the results, but we do not have data on who received it. Studies have shown that psychological debriefing is ineffective and can even be harmful [34]. We cannot rule out that some of the participants may have received debriefing, which may have affected their symptom levels. Nevertheless, the high symptom levels in the current study are more likely to be influenced by other factors, aforementioned, such as recurrent media debates and different official prolonged investigations. Despite these limitations, a particular strength of the study is its longitudinal design with 7 time-points as well as the large sample size.

## Conclusions

The present study suggests that bereavement in disaster can lead to high levels of PTS which are sustained for several years, even though the individuals themselves have not been directly exposed to the disaster. A remarkably high proportion of individuals, i.e. 90%, scored above the cut-off value in the group who had lost a child of any age in the disaster in the three-year follow-up. In general, individuals with initially high levels of PTS were associated with a slower recovery rate. Women showed initially more PTS but they recovered more quickly than men. Younger respondents showed a steeper decrease in PTS over time than older respondents who recovered at a slower rate. This study highlights the importance of considering how the traumatically bereaved can be enormously affected by indirect exposure to disaster.
